# Validity and reliability of a Malay version of the brief illness perception questionnaire for patients with type 2 diabetes mellitus

**DOI:** 10.1186/s12874-017-0394-5

**Published:** 2017-08-03

**Authors:** Boon-How Chew, Rimke C. Vos, Monique Heijmans, Sazlina Shariff-Ghazali, Aaron Fernandez, Guy E. H. M. Rutten

**Affiliations:** 1 Department of Family Medicine, Faculty of Medicine and Health Sciences, Universiti Putra Malaysia, 43400 Serdang, Selangor Malaysia; 2 Julius Center for Health Sciences and Primary Care, Department of General Practice, University Medical Center Utrecht, Huispost Str.6.131, P.O. Box 85500, 3508 GA Utrecht, The Netherlands; 3 NIVEL, Netherlands Institute for Health Services Research, Utrecht, Netherlands; 4 Department of Psychiatry, Faculty of Medicine and Health Sciences, Universiti Putra Malaysia, 43400 Serdang, Selangor Malaysia

**Keywords:** Beliefs, Brief illness perception questionnaire, Validity, Illness perceptions, Type 2 diabetes mellitus, Self-efficacy, Medication adherence, Quality of life, Emotional distress, HbA1c

## Abstract

**Background:**

Illness perceptions involve the personal beliefs that patients have about their illness and may influence health behaviours considerably. Since an instrument to measure these perceptions for Malay population in Malaysia is lacking, we translated and examined the psychometric properties of the Malay version of the Brief Illness Perception Questionnaire (MBIPQ) in adult patients with type 2 diabetes mellitus.

**Methods:**

The MBIPQ has nine items, all use a 0–10 response scale, except the ninth item about causal factors, which is an open-ended item. A standard procedure was used to translate and adapt the English BIPQ into Malay language. Construct validity was examined comparing item scores and scores on the Diabetes Management Self-Efficacy Scale, the Morisky Medication Adherence Scale, the World Health Organization Quality of Life-brief, the 9-item Patient Health Questionnaire, the 17-item Diabetes Distress Scale, HbA1c and the presence of complications. In addition, 2-week and 4-week test-retest reliability were studied.

**Results:**

A total of 312 patients completed the MBIPQ. Out of this, 97 and 215 patients completed the 2- or 4-weeks test-retest reliability questionnaire, respectively. Moderate inter-items correlations were observed between illness perception dimensions (*r* = −0.31 to 0.53). MBIPQ items showed the expected correlations with self-efficacy (*r* = 0.35), medication adherence (*r* = 0.29), quality of life (*r* = −0.17 to 0.31) and depressive symptoms (*r* = −0.18 to 0.21). People with severe diabetes-related distress also were more concern (t-test = 4.01, *p* < 0.001) and experienced lower personal control (t-test = 2.07, *p* = 0.031). People with any diabetes-related complication perceived the consequences as more serious (t-test = 2.04, *p* = 0.044). The 2-week and 4-week test-retest reliabilities varied between ICC_agreement_ 0.39 to 0.70 and 0.58 to 0.78, respectively.

**Conclusions:**

The psychometric properties of items in the MBIPQ are moderate. The MBIPQ showed good cross-cultural validity and moderate construct validity. Test-retest reliability was moderate. Despite the moderate psychometric properties, the MBIPQ may be useful in clinical practice as it is a useful instrument to elicit and communicate on patient’s personal thoughts and feelings. Future research is needed to establish its responsiveness and predictive validity.

**Trial registration:**

ClinicalTrials.gov NCT02730754 registered on March 29, 2016; NCT02730078 registered on March 29, 2016.

**Electronic supplementary material:**

The online version of this article (doi:10.1186/s12874-017-0394-5) contains supplementary material, which is available to authorized users.

## Background

Illness perceptions refer to the personal beliefs that patients have about their illnesses [1, 2]. Among others, illness perceptions constitute beliefs about the typical complaints that belong to the illness, beliefs about the course, the consequences and the extent to which a disease is controllable either by self-care or medical care; they also include the understanding a patient has of the illness [2]. Illness perceptions evaluate the emotional impact of the illness directly and indirectly from the symptoms experienced and concern about the illness’s consequences [3]. Illness perceptions are an important determinant of health behaviours such as in treatment adherence and via health behaviour they indirectly influence outcomes such as quality of life, functional recovery and clinical parameters [4]. Intervention studies have shown that illness perceptions can be changed [2, 5].

In both type 1 and type 2 diabetes mellitus (T2DM) patients illness perceptions have an independent effect on dietary management, physical exercise, self-monitoring of blood glucose, medication adherence, foot care, smoking cessation, appointment attendance, anxiety and depression [6]. Croatian adults with T2DM who reported a better illness understanding and experienced more personal control had a healthier lifestyle than those with more negative illness perceptions [7]. Some illness perceptions, namely beliefs about personal control and understanding, were significantly associated with glycaemic control [2, 8].

People who hold stronger beliefs that their diabetes is chronic and with serious consequences reported a higher emotional impact of their disease; lower perceived personal control was associated with increased depression and anxiety in both type 1 and 2 diabetes patients [6]. Perceived control over diabetes partially mediated the relationship between diabetes-related distress and poorer treatment adherence and glycaemic control among adults with T2DM [9]. The perceived impact of diabetes 4 months after the diagnosis remained a significant predictor of distress and depression at 3-year follow-up [10]. Patients’ perception of their illnesses and related symptoms and their beliefs about the possible consequences of the disease are also associated with their satisfaction with medical consultation and healthcare utilisation, respectively [11].

Malaysian data showed an increase in the prevalence of T2DM, a high number of diabetes-related complications and persistent poor disease control and management among T2DM patients [12, 13], especially among the Malays [14]. Given the importance of illness perceptions for health behaviour and outcomes and given the rising burden of T2DM and its associated mood disorders in Malaysia [15, 16], we translated and validated a Malay version of the Brief Illness Perception Questionnaire (MBIPQ) to facilitate the assessment of illness perceptions in a Malay-speaking population. In this study three questions are addressed:

1. What is the cross-cultural validity and construct validity of the MBIPQ in Malay-speaking patients with T2DM?
2. What is the discriminant validity (hypotheses testing) of the MBIPQ in relation to glycaemic control, diabetes-related distress and diabetes-related complications?
3. What is the intrarater test-retest reliability of the MBIPQ in clinical practice?

The first two questions are about the construct validity and the third question is about reliability of MBIPQ [17]. These three questions are chosen because they are important and fundamental when deciding on using the MBIPQ in practice and before further testing on its other psychometric properties such as responsiveness. Since the BIPQ is considered to be a multiple single-item measures assessing different aspects of a complex phenomenon, namely perception of diabetes (see below), its factor structure, item-total correlation or internal consistency are considered to be less relevant and usually not tested [18]. In most studies, the original English version of BIPQ has only been on test-retest reliability, discriminant validity, predictive validity and concurrent validity with the more extensive Illness Perception Questionnaire-Revised (IPQ-R) [19], and not on the factor structure or its dimensionality using the item response theory or Rasch approaches [18]. Therefore, we also decided not to explore the internal consistency or factor structure of the MBIPQ. Since content and face validity of BIPQ was already established, we also decided not to describe content and face validity in this study as we translated an existing and fully developed BIPQ. Instead, we focus on cross-cultural validity, construct validity, discriminant validity and intrarater test-retest reliability of each item [17].

## Methods

### The 9-item brief illness perception questionnaire (BIPQ)

The BIPQ was designed to provide simple and rapid assessment of illness perceptions. It was developed for use in clinical practice and consists of nine items. These nine items are as below, and available at http://www.uib.no/ipq/pdf/B-IPQ-English.pdf (Additional file 1). Like the IPQ-R, the general version of the BIPQ uses the word ‘illness’, but it is possible to replace this with the name of a particular illness such as T2DM or diabetes mellitus and this was done for the MBIPQ.

1. (Consequences) How much does your diabetes affect your life?
2. (Timeline) How long do you think your diabetes will continue?
3. (Personal Control) How much control do you feel you have over your diabetes?
4. (Treatment Control) How much do you think your treatment can help your diabetes?
5. (Identity) How much do you experience symptoms from your diabetes?
6. (Concern) How concerned are you about your diabetes?
7. (Understanding) How well do you feel you understand your diabetes?
8. (Emotional Response) How much does your diabetes affect you emotionally? (e.g. does it make you angry, scared, upset or depressed)?
9. (Causal Representation) Please list in rank-order the three most important factors that you believe caused your diabetes. The most important causes for me:- 1)__2)__3)__

All items were developed by forming one question that best summarised the items contained in each subscale of the IPQ-R [20]. Five items assess cognitive illness representations: perceived consequences (Item 1), timeline (acute-chronic) (Item 2), personal control (Item 3), treatment control (Item 4), and the presence of symptoms or identity (Item 5). Two items assess emotional representations: concern (Item 6) and emotions (Item 8) about the illness. One item assesses illness comprehensibility or coherence of the illness (Item 7). In contrast to the more traditional method of constructing dimensions by forming subscales, the BIPQ has just one single item to assess each dimension, rated on a 0-to-10 scale like in the IPQ-R. Higher scores indicate stronger perceptions along that dimension. The BIPQ has been shown to have good psychometric properties in 36 countries and many illness populations [2]. The test-retest reliability correlation coefficients of the original English BIPQ were between 0.48 to 0.70 at 3-weeks and 0.42 to 0.75 at 6-weeks [19]. The equivalent scales of the BIPQ and the IPQ-R are moderately correlated (0.32 to 0.62), including the causal representation item with 75% of all causes categorised within the 20 causal factors [19]. The personal control item was significantly correlated with diabetes self-efficacy (*r* = 0.61, *p* < 0.001) and lower HbA1c (*r* = −0.30, *p* < 0.01) [19]. In a systematic review in 2015 [2], pooled correlations were undertaken based on the Fisher’s z transformation of the correlation coefficients, showed that between illness perceptions and depression, blood glucose levels and quality of life were in the range 0.25 to 0.49 for consequences, identity and emotional representations and between - 0.15 to - 0.27 for the personal control item. All items were able to predict some outcomes up to one-year follow-up [2]. Personal control and causal items showed most frequent changes after intervention in randomised controlled trials [2].

Besides these single items, the BIPQ has a part of the causal scale previously used in the IPQ-R [19]. Assessment of the causal representation is by an open-ended response item, which asks patients to list the three most important causal factors of their illness (Item 9). Because each item of the BIPQ assesses one dimension of illness perceptions, the consequences score is simply the response to item 1 et cetera. Reponses to item 9 can be grouped into categories such as lifestyle (food and physical exercise), hereditary, stress, etc.

### Setting

This study was conducted in 2016 in 11 public health clinics in Malaysia, three in Selangor and eight in Negeri Sembilan. These are governmental health clinics in urban, suburban and rural areas that have resident doctors and are headed by family medicine specialists. They provide primary medical care collaborating with a multi-disciplinary team of a nutritionist or dietician, pharmacist, physiotherapist, occupational therapist and paramedics who have undergone specialised training in diabetes education and eye care [21].

### Study samples

We used the data of 312 patients with T2DM for this study. They came from two different samples. This number is considered sufficient as the required sample size for a validation study is the number of items × 10 [18, 22]. Patients were included in the study according to the following criteria: patients who understand Malay, at least 30 years old, diagnosed with T2DM for at least 3 years and with a regular follow-up with three or more visits in the past year. Patients who were pregnant or breastfeeding, or those with severe health problems or psychiatric/psychological disorders that caused cognitive impairments or those who cannot self-administer or be interviewed to complete the questionnaires were excluded. Severe health problems such as life-threatening diseases, recent acute complications or injuries and a recent discharge from hospital comprised the other exclusion criteria. The definition of T2DM was based on: (i) a documented diagnosis of diabetes mellitus according to the World Health Organization criteria or (ii) current treatment consisting of lifestyle modification, oral anti-hyperglycaemic agents or insulin. Approval was obtained from the original author to use the BIPQ English version. All subjects had provided written informed consent before participation.

### Instrument translation and cross-cultural validation

**Fig. 1:**
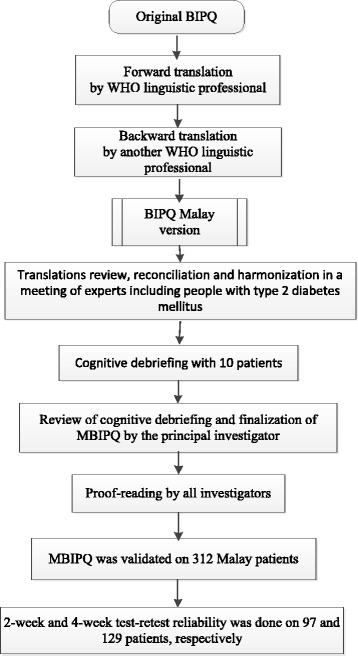
Translation and validation process. BIPQ = Brief Illness Perception Questionnaire. MBIPQ = Malay version of the Brief Illness Perception Questionnaire. WHO = World Health Organization

The translation and adaptation process of the BIPQ from its original language (English) to Malay is shown in Fig. 1. It was conducted according to guidelines for cross-cultural adaption of self-report measures [23, 24]. The cultural adaptation process included: (1) review of the original and the two translated questionnaires by an expert committee that was composed of researchers, three family medicine specialists, a psychologist, a methodologist, and three adults with T2DM; except one researcher and the methodologist, the main researcher, the three family medicine specialists, the psychologist, and three adults with T2DM were bilingual (Malay and English) and the three family medicine specialists and the three adults with T2DM were native language (Malay) speakers; (2) reconciliation, (3) harmonisation, (4) cognitive debriefing, (5) review of cognitive debriefing results and finalisation, (6) proofreading and (7) final report.

This version of the MBIPQ was distributed to 10 Malay patients with the above mentioned inclusion and exclusion criteria for comment. These individuals were not included in the later validation study. They commented that the items were easily understood and acceptable with regards to their cultural background. Therefore, that MBIPQ version was accepted without modification for the validation study.

### Assessing construct validity

Construct validity for the eight items in MBIPQ, as a measure of different illness perceptions, was assessed by comparing self-efficacy, quality of life, medication adherence, diabetes-related distress and depressive symptoms with each item of the MBIPQ. For that purpose, patients self-administered the socio-demographic and MBIPQ questionnaires, and for those who could not, face-to-face interviews were performed by trained research assistants. Additionally, they completed a set of the below mentioned five questionnaires:

1. The 20-item Diabetes Management Self Efficacy Scale (DMSES) measures patients’ confidence in managing their disease in terms of blood glucose, diet, and exercise, with items on an 11-point Likert scale, scores ranging from 0 if they “cannot do at all” to 10 if the respondents “certainly can do”; lower scores indicating low self-efficacy for coping with the activities listed [25–27]. In social cognitive theory of self-regulation [28, 29], self-efficacy plays important roles in health behaviours through its influences on personal thoughts, emotion, motivation and action. Previous research has shown significant moderate correlations between diabetes self-efficacy and perceived control (*r* = 0.61) [19], and we expected to find similar correlations between the MBIPQ personal control item and self-efficacy.
2. The World Health Organization Quality of Life-brief version (WHOQOL-BREF), 25 items measuring quality of life over the past 4 weeks in the physical, psychological, social relationships and environmental domain, with scores from 25 to 100 and higher scores denoting higher quality of life) [30–32]. WHOQOL-BREF assesses activities of daily living, thinking, learning, environment safety, freedom to move about, feelings and dependence on medical therapies which are influenced by health beliefs and personal illness perception as measured by MBIPQ. Previous studies showed that higher consequences (*r* = 0.37 to 0.49), emotional representation and identity (*r* = 0.34 to 0.43) were associated with lower quality of life; and personal control (*r* = 0.18 to 0.22) and treatment control (*r* = 0.16) had significant correlations with better quality of life [2]. We expected to find similar correlations in MBIPQ with this quality of life measure.
3. The 8-item Morisky Medication Adherence Scale (MMAS-8), measuring medication adherence during the past 2 weeks, and with scores from 0 to 8, resulting in the following levels of adherence: low (< 6), medium (6,7) and high (8) [33–36]. MMAS-8 allows a construct assessment of the treatment control item in MBIPQ [37, 38]. Based on literature, we expected to find a positive correlation (approximately *r* = 0.20) between a higher belief in treatment as well as a higher perception of personal control on the one hand and medication adherence on the other [39, 40].
4. The 17-item Diabetes Distress Scale (DDS-17) assesses problems and hassles concerning diabetes during the last month. Each item is scored on a Likert scale from 1 (not a problem) to 6 (a very serious problem). It yields a total scale score with a mean total score of ≥3 (severe distress) is considered a level of distress worthy of clinical attention [41, 42]. Previous studies showed that distress influences perceptions of self-management and were associated with poor diabetes-treatment adherence and HbA1c [9], lower self-efficacy, and poorer dietary and exercise behaviours [43–45]. Examining the correlations between the emotional items of the MBIPQ with diabetes-related distress as measured with the DDS-17 in order to test the construct validity of the MIBQ, we expected to find correlations of approximately *r* = 0.40 between both the items ‘consequences’, ‘concern’ and ‘emotional response’ on the one hand and diabetes-related distress [46].
5. The 9-item Patient Health Questionnaire (PHQ-9) [47, 48] evaluates depressive symptoms and grades the depression severity. It scores from 0 to 27, which can be classified as 0–4 (minimal), 5–9 (mild), 10–14 (moderate), 15–19 (moderately severe), and 20–27 (severe) [47, 48]. Previous studies with the BIPQ showed that higher consequences (*r* = 0.41), emotional response (*r* = 0.39) and identity (*r* = 0.32) were associated with higher depression; personal control (*r* = −0.15) and treatment control (*r* = −0.11) had small negative correlations with depression [2]. We expected to find similar correlations between MBIPQ with depressive symptoms.

### Discriminant validity

The discriminant validity of the MBIPQ was examined in relation to HbA1c, an estimate of blood glucose control over the past 3 months; and also by its ability to distinguish between patients with severe and non-severe diabetes-related distress (mean DDS-17 score ≥ 3 versus <3) [43] and between those with and without diabetes-related complications. HbA1c results within the past 3 months were retrieved from medical records. This is analysed in the respective health clinics or regional hospitals with government approved analysers that are calibrated on annual basis. Diabetes-related complications were also retrieved from the medical records, the diagnoses of these conditions are based on clinical practice guidelines.

### Intrarater test–retest reliability

To examine the test–retest reliability, patients in the eight public health clinics in Negeri Sembilan were invited for a 2-week test–retest and those in the three Selangor public health clinics for a 4-week re-test, respectively. The former were participants in a trial that had an interval of 2 weeks between their baseline meeting and first session of the programme, the latter would return to the clinic for medication re-supply. Data collection procedures were similar and standardised between test and retest. Participants in Negeri Sembilan answered the MBIPQ before the first session of the programme, whereas those in Selangor completed MBIPQ in the waiting area of the health clinics while waiting for a doctor’s consultation.

### Statistical analyses

Descriptive statistics were used to describe the demographic and disease characteristics of the patients and their MBIPQ item scores. The mean (SD) scores of the first eight items of the MBIPQ were reported at the first, the 2-week and 4-week testing. Floor and ceiling effects at each testing moment were also reported. To assess the construct validity of the MBIPQ the following analyses were performed: Spearman’s or Pearson’s correlation coefficients were calculated and Student t-tests were carried out for MBIPQ item scores with self-efficacy, quality of life, medication adherence, diabetes-related distress and depressive symptoms as categorical and continuous independent variables, respectively. The discriminant validity of the MBIPQ was first examined by measuring the Pearson’s or Spearman’s between each item of the MBIPQ and HbA1c. To distinguish between patients with severe and non-severe diabetes-related distress and between those with and without diabetes-related complications, Student t-tests or the Mann-Whitney U test were performed.

Pearson’s correlations were calculated for normally distributed and for non-normally distributed variables (items/scales) if Spearman’s correlations gave similar results. Otherwise, Spearman’s correlations were reported. Comparing the associations of severe / non-severe diabetes-related distress, and of diabetes-related complication / no complication with all the MBIPQ item scores, the Student t-tests and the Mann-Whitney U test showed similar results (except item 3). We report the association with Student t-test. Correlations were interpreted using the following Cohen’s criteria: 0–0.25 = little or no correlation, 0.25–0.5 = fair correlation, 0.5–0.70 = moderate to good correlation and >0.70 = very good to excellent correlation [49].

Since the MBIPQ uses a single-item scale approach to assess perceptions on a continuous linear scale, we used intraclass correlation coefficients (ICC_agreement_) of two-way random effects model to assess test–retest reliability [18] at 2-week and 4-week. This will take into account systematic differences that Pearson’s or Spearman’s correlation does not [18]. ICC_agreement_ of 0.70 is recommended as a minimum standard for reliability [18]. Besides, the three most common causes for T2DM in item 9 of the MBIPQ were presented. As these causes were ordinal and ranked, we used Spearman’s correlation to examine the correlation of the causes that were ranked as first, second and third between the first and the repeated responses at 2-week and 4-week. Statistical analyses were performed using Statistical Package for Social Science 22.0 (SPSS, Chicago, IL). The significance level was set at *p* < 0.05.

## Results

A total of 324 patients (100 of 123 from Negeri Sembilan and 224 of 361 from Selangor) with T2DM responded but 312 patients (97 of 100 from Negeri Sembilan and 215 of 224 from Selangor) completed the MBIPQ. The age of the non-responders was significantly older than the participants (61.7 vs. 58.2 years, respectively, t test = 3.28, *p* = 0.001) and gender (male sex 44.4 and 42.3%, respectively, χ^2^ = 0.18, *p* = 0.680) was not significantly different between the two groups. All the 97 patients from Negeri Sembilan completed the 2-week test-retest and 129 patients from Selangor completed the test–retest after 4 weeks. The demographic characteristics of the participants are presented in Table 1.

**Table 1:** Sample characteristics

	Validation, *n = 312*	Negeri Sembilan 2-week test-retest reliability, *n = 97*	Selangor 4-week test-retest reliability, *n = 129*
Age, mean (SD)*	58.1 (9.67)	56.1 (9.10)	59.1 (9.61)
Diabetes duration in years, mean (SD)	8.5 (4.79)	8.5 (5.92)	8.3 (4.15)
	n (%)		
Gender			
Female	184 (59.0)	61 (62.9)	69 (53.5)
Male	128 (41.0)	36 (37.1)	60 (46.5)
Ethnicity^a^			
Malay	240 (76.9)	97 (100)	87 (67.4)
Chinese	9 (2.9)	0	9 (7.0)
Indian	61 (19.6)	0	32 (24.8)
Educational level^a^			
No school	15 (4.9)	0	9 (7.1)
Primary school level	99 (32.2)	14 (14.6)	55 (43.3)
Secondary school level	161 (52.4)	70 (72.9)	56 (44.1)
Tertiary school level	32 (10.4)	12 (12.5)	7 (5.5)
Life event in the past 6 months^a^			
Yes	61 (19.7)	27 (28.4)	16 (12.4)
No	248 (80.3)	68 (71.6)	113 (87.6)
Co-morbidity			
Hypertension	269 (86.2)	84 (86.6)	108 (83.7)
Dyslipidaemia	256 (82.1)	84 (86.6)	107 (82.9)
Any diabetes complication^a^	98 (31.4)	25 (25.8)	51 (39.5)
Treatment			
Oral hypoglycaemic agent^a^	266 (85.3)	94 (96.9)	103 (79.8)
Insulin^a^	161 (51.6)	74 (76.3)	58 (45.0)
HbA1c in %, mean (SD)^a^	9.0 (2.27)	9.7 (1.99)	8.9 (2.43)

*SD* standard deviation

^a^ Statistically significant differences between the Negeri Sembilan and Selangor at *p* value <0.05

### Construct validity

Table 2 shows the mean (SD) of the eight items in MBIPQ at each testing. Floor effects, with more than 15% of the participants mentioning the lowest possible score [18] were seen in the item understanding (19.4%) at the first assessment. The ceiling effects, with more than 15% of the participants mentioning the highest possible score [18] were seen in the items timeline (35.3%) and concern (26.1%) at the first assessment, and again in item timeline (27.7%) at the 4-week retest.

**Table 2:** Mean (SD) scores on eight items of the Malay Brief Illness Perception Questionnaire at each testing

MBIPQ Item (Min. - Max.)	0 week	2 weeks	4 weeks
Consequences (0–10)	4.7 (2.63)	4.4 (2.54)	5.0 (2.14)
Timeline (0–10)	7.2 (2.86)	6.2 (2.42)	8.2 (2.29)
Personal Control (0–10)	2.9 (2.07)	3.1 (2.13)	3.0 (1.73)
Treatment Control (0–10)	1.8 (1.88)	2.4 (2.09)	1.6 (1.79)
Identity (0–10)	4.8 (2.61)	5.0 (2.28)	5.3 (2.08)
Concern (0–10)	6.9 (2.87)	6.4 (2.67)	6.7 (2.43)
Understanding (0–10)	2.6 (2.22)	2.7 (2.08)	2.9 (1.85)
Emotional Response (0–10)	5.2 (2.82)	5.2 (2.57)	5.3 (2.49)

Table 3 shows that illness perceptions correlated in a logical way, at best moderate: perceiving symptoms (identity) was significantly related to being affected emotionally by the diabetes (emotional response) (*r* = 0.53) and being concerned about their T2DM was related to perceiving consequences of the disease (*r* = 0.42). Significant correlations were also noted between perceived personal (*r* = 0.31) as well as treatment control (*r* = 0.35) and understanding of the illness. Conversely, those who understood more about their T2DM also expressed lesser concern (*r* = −0.30). Negative correlations between timeline and perceived personal control (*r* = −0.23), and timeline and perceived treatment control (*r* = −0.21), indicated that perception of chronicity was associated with loss of perceived personal control and treatment ineffectiveness, respectively.

**Table 3:** Items inter-correlations (Pearson’s correlation coefficients) of the Malay Brief Illness Perception Questionnaire in adults with type 2 diabetes mellitus

	Pearson’s correlation, *r* (n)							
	Consequences	Timeline	Personal Control	Treatment Control	Identity	Concern	Understanding	Emotional Response
Consequences	1 (324)							
Timeline	0.233^**^ (320)	1 (320)						
Personal Control	0.090 (322)	−0.231^**^ (318)	1 (322)					
Treatment Control	0.082 (322)	−0.211^**^ (319)	0.505^**^ (320)	1 (322)				
Identity	0.503^**^ (321)	0.259^**^ (318)	0.072 (319)	0.016 (320)	1 (321)			
Concern	0.418^**^ (322)	0.131^*^ (318)	−0.042 (320)	−0.058 (320)	0.379^**^ (319)	1 (322)		
Understanding	−0.087 (324)	−0.142^*^ (320)	0.314^**^ (322)	0.352^**^ (322)	−0.060 (321)	−0.303^**^ (322)	1 (324)	
Emotional Response	0.525^**^ (322)	0.138^*^ (318)	0.009 (320)	0.001 (320)	0.532^**^ (320)	0.514^**^ (320)	−0.165^**^ (322)	1 (322)

^*^ *p* value <0.05, ^**^ *p* value <0.01

Overall, there were 71 and 141 participants who did not provide the cause for their T2DM in the first and repeated MBIPQ, respectively. The three most common causes for T2DM listed by the participants in the first MBIPQ were dietary (283/603), sedentary lifestyle (114/603) and hereditary (108/603). Other causes (98/603) mentioned at least 10 times included obesity (16), emotional conditions such as stress (16), lack of health-related knowledge (16), medication (10) and lifestyle factor (10). There were significant correlations of the first (*r* = 0.35, *p* = 0.003, *n* = 70), second (*r* = 0.30, *p* = 0.031, *n* = 53) and third (*r* = 0.37, *p* = 0.021, *n* = 38) important causes as mentioned in the 2-week interval between the first and second measurement. However, there were no significant correlations noted for all three important causes in the 4-week interval.

Table 4 shows a number of correlations between MBIPQ items and patient-reported outcomes. Some correlations were in the expected range of approximately 0.20, namely three out of eight with self-efficacy, two with medication adherence, five with quality of life, five with depressive symptoms and seven with diabetes-related distress. This indicates a fair construct validity (hypotheses testing) of the MBIPQ. Those who understood their T2DM well or had a higher perception of personal or treatment control had a higher self-efficacy. Similarly, patients who had a higher personal control or perceived treatment effectiveness according to the MBIPQ were more adherent to their medication, experiencing better quality of life, a lower HbA1c and less depressive symptoms. Significant correlations were also noted between item Identity (experiencing diabetic symptoms) and less self-efficacy, lower quality of life and more depressive symptoms.

**Table 4:** Correlation (Pearson’s correlation coefficients) between MBIPQ and self-efficacy, medication adherence, quality of life, depressive symptoms, diabetes-related distress and HbA1c

No.	MBIPQ Item	Self-efficacy	Medication adherence	Quality of life	Depressive symptoms	Total DDS-17	HbA1c
1.	Consequences	−0.05	−0.09	−0.17^**^	0.21^**^	0.28^**^	0.10
2.	Timeline	0.10	0.10^a^	0.05	−0.11^b^	−0.15^**^	−0.09
3.	Personal Control	0.35^**^	0.23^**^	0.31^**^	−0.12^*^	−0.23^**^	−0.13^*^
4.	Treatment Control	0.26^**^	0.29^**^	0.31^**^	−0.18^**^	−0.17^**^	−0.16^**^
5.	Identity	−0.13^*^	−0.04	−0.17^*^	0.16^**^	0.23^**^	0.09
6.	Concern	0.05	−0.04	−0.09	0.21^**^	0.28^**^	0.23^**^
7.	Understanding	0.35^**^	−0.06	0.14^*^	−0.03	0.004	0.12^*^
8.	Emotional Response	−0.02	−0.10	−0.18^**^	0.29^**^	0.28^**^	0.15^**^

*MBIPQ* Malay version of the Brief Illness Perception Questionnaire, *DDS-17* the 17-item Diabetes Distress Scale

^*^ *p* value <0.05, ^**^ *p* value <0.01

^a^ Spearman correlations was 0.15 at *p* = 0.010

^b^Spearman correlations was −0.20 at *p* < 0.01

### Discriminant validity

Patients who had a higher personal control or perceived treatment effectiveness according to the MBIPQ had a lower HbA1c (Table 4). The relationship between the MBIPQ items and the total diabetes-related distress score is also depicted in Table 4. Those who had higher diabetes-related distress were generally having a threatening view about T2DM (except understanding). Those who had severe diabetes-related distress (DDS-17 scores ≥3) were perceiving more consequences (t-test = 3.20, *p* = 0.002), identity (t-test = 2.97, *p* = 0.003) and concern (t-test = 4.01, *p* < 0.001), and lower personal control (t-test = 2.07, *p* = 0.031). Perceiving the diabetes treatment not effective (t-test = 2.39, *p* = 0.017) was also associated with more severe diabetes-related distress. Patients with and any diabetes-related complication were also having higher consequences (t-test = 2.04, *p* = 0.044) and identity scores (t-test = 2.86, *p* = 0.005).

### Reliability

Table 5 shows the intrarater test-retest correlations after 2- and 4-week. The 2-week test–retest reliability ICC_agreement_ values ranged from 0.39 to 0.70. The respective values after 4-weeks are 0.58 to 0.78, with the highest for concern.

**Table 5:** Intraclass correlation coefficients of the Malay Brief Illness Perception Questionnaire

MBIPQ Item, (n)	2 weeks, (n)	4 weeks, (n)
Consequences (*n* = 324)	0.677^**^ (*n* = 100)	0.680^**^ (*n* = 133)
Timeline (*n* = 320)	0.697^**^ (*n* = 98)	0.582^**^ (*n* = 133)
Personal Control (*n* = 322)	0.387^*^ (*n* = 99)	0.685^**^ (*n* = 133)
Treatment Control (*n* = 322)	0.569^**^ (*n* = 100)	0.671^**^ (*n* = 133)
Identity (*n* = 321)	0.498^**^ (*n* = 100)	0.664^**^ (*n* = 132)
Concern (*n* = 322)	0.687^**^ (*n* = 100)	0.781^**^ (*n* = 131)
Understanding (*n* = 324)	0.462^**^ (*n* = 100)	0.664^**^ (*n* = 132)
Emotional Response (*n* = 322)	0.652^**^ (*n* = 100)	0.610^**^ (*n* = 132)

^*^ *p* value <0.05, ^**^ *p* value <0.01

## Discussion

MBIPQ provides a rapid and moderately good assessment of the personal beliefs that patients hold about their T2DM. Thus, MBIPQ may be of value for clinical practice as a starting point in clinical consultations to talk about patient’s worries about T2DM, feeling overwhelmed and beliefs in treatment options. This would allow a more tailored treatment and support from the healthcare team. This study provided a translated and culturally adapted Malay version of the BIPQ. Inter-items correlations were at best moderate, and correlations with the studied patient-reported outcomes were at best fair according to the Cohen’s criteria. The discriminant validity of the MBIPQ was supported by its ability to distinguish between patients with different levels of diabetes-related distress and diabetes-related complications. The intrarater test-retest reliability was good for a few items but less than the recommended standard [18] between most items.

MBIPQ has moderate construct validity as some items showed the expected hypothesised correlation coefficients with the patient-reported outcome measures. Correlations between the MBIPQ treatment and personal control items and self-efficacy, medication adherence, quality of life and emotional distresses are in line with those of former studies [2, 39, 40]. The correlation coefficient between perceived personal control and diabetes self-efficacy was about half of that observed in the original English BIPQ study which was measured with the Multidimensional Diabetes Questionnaire (*r* = 0.61, *p* < 0.001) [19]. Other cognitive perceptions also showed the expected association with medication adherence indicating that those who perceived and experienced T2DM in a more positive manner also tend to adhere to their medication. We could not demonstrate significant associations between timeline with quality of life and depression, in contrast to a meta-analysis [2] that showed that a higher perception of chronicity of diabetes was associated with on the one hand better quality of life, but on the other a higher depression rate, more anxiety and higher HbA1c. However, the association between timeline and diabetes-related distress was negative, indicating that those who believe that T2DM is of short duration are experiencing higher diabetes-related distress. It is difficult to explain this association in this study although age could be the confounding factor [15, 50]. It seems plausible that those who experienced much symptoms (identity) expressed lower self-efficacy, lower quality of life and more depressive symptoms. The same applies to MBIPQ items that showed expected results with the diabetes-related distress. Patients who perceived better personal or treatment control demonstrated less depressive symptoms and less diabetes-related distress while those who were distressed in this respect showed high concern and emotional response to T2DM. The associations between perceptions of better personal or treatment control and less depressive symptoms and a lower HbA1c are in concordance with earlier studies [2, 51]. Our finding that more concern and emotional perceptions were significantly associated with higher HbA1c are in line with the results of previous studies [51]. However, in contrary to past studies MBIPQ did not show consequences and identity beliefs to have any significant correlation with HbA1c [2].

Intraclass correlation coefficients were almost all below 0.70. In our opinion this does not mean that the MBIPQ is an unreliable measure. Illness perceptions as assessed by the MBIPQ are susceptible to change, for example after an intervention [2]. Sensitivity to change of the MBIPQ needs to be studied further. The similar or even slightly better 4-week test-retest reliability compared to the 2-week reliability test might be due to the two different cohorts of respondents in this study. The people from Negeri Sembilan experienced more life events in the past 6 months (Table 1) and had more emotional distresses [52]; these differences might have led to fluctuation in illness perceptions through impairment of self-management ability [53] and cognitive function [54]. Obviously, this will result in lower intrarater test-retest correlations, as we found. Because we used ICC_agreement_ for the test-retest, it is hard to compare the test-retest reliability with the results of the other BIPQ versions that calculated Pearson’s correlation such as the Chinese version (*r* = 0.24 to 0.76) and the Farsi version (*r* = 0.50 to 0.75) [8] that were retested after 4 weeks [55], the original English version that was retested after 3 weeks (*r* = 0.48 to 0.70) and 6 weeks (*r* = 0.42 to 0.75) [19]. Nevertheless, the test-retest reliability of the MBIPQ is found to be satisfactory and more or less comparable to the other versions [56, 57].

### Strength and limitations

Standard translation with rigorous adaptation procedures by an expert committee that included patients with T2DM had produced the MBIPQ of good cross-cultural validities. Further, adequate sample size and variety of the respondents were the strength of this study. Construct and discriminant validation were done with multiple validated and well known outcomes. However, having nine items instead of one index or summary score to handle in statistical models may be a major limitation of (M)BIPQ. We could not handle MBIPQ as a single construct (a reflective model in which all items are a manifestation of the same underlying construct) because of the low inter-items correlations in our study population. We may consider the MBIPQ as a formative measure if we assume that all item scores will change after for example an education on diabetes self-management [58]. In addition, adding up items gets information lost about which perceptions are most strongly linked to the studied outcomes such as between personal control and understanding and self-efficacy, between treatment control and medication adherence, etc. (data not shown). Another limitation is the lack of equivalent scales (such as knowledge on T2DM) for some of the illness perception dimensions which hinders a full investigation of MBIPQ construct validity. Due to the inclusion criteria the results of this study are less applicable to the Chinese community in Malaysia and to T2DM patients who are treated in a secondary care setting. Future research is needed to establish its properties on responsiveness and predictive validity.

## Conclusions

The psychometric properties of items in the MBIPQ are moderate. The MBIPQ showed good content and face validity. Construct validity showed small but significant correlations for all illness perception dimensions with relevant outcome measures, and test-retest reliability was generally moderate. The discriminant validity of the MBIPQ was supported by its ability to distinguish between categories of patients with different categories of diabetes-related distress and diabetes related complications. For that reason we think the MBIPQ may be useful in clinical practice, it offers an opportunity to assess and potentially modify people’s perceptions, understanding and experience of T2DM. However, the timeline and concern items do not perform well and should be used with most caution.

## Additional file

Additional file 1: Two questionnaires. The first one is the Malay version of the Brief Illness Perception Questionnaire (BIPQ), and the second one is the English translation of the BIPQ. (PDF 89 kb)

## Abbreviations

BIPQ: The 9-item Brief Illness Perception Questionnaire
DDS-17: 17-item Diabetes Distress Scale
DMSES: Diabetes Management Self Efficacy Scale
IPQ-R: Illness Perception Questionnaire-Revised
MBIPQ: Malay version of the Brief Illness Perception Questionnaire
MMAS-8: 8-item Morisky Medication Adherence
PHQ-9: 9-item Patient Health Questionnaire
T2DM: Type 2 diabetes mellitus
WHOQOL-BREF: World Health Organization Quality of Life-brief
