# Supplementary material for: Validity and reliability of a Malay version of the brief illness perception questionnaire for patients with type 2 diabetes mellitus
Source: BMC Med Res Methodol. 2017 Aug 3;17:118. doi: 10.1186/s12874-017-0394-5 (PMC5543429; doi:10.1186/s12874-017-0394-5)
Supplement: Additional file 1: — Two questionnaires. The first one is the Malay version of the Brief Illness Perception Questionnaire (BIPQ), and the second one is the English translation of the BIPQ. (PDF 89 kb) [file 12874_2017_394_MOESM1_ESM.pdf]

Soal Selidik Ringkas Persepsi Penyakit (MBIPQ) ©

Bil.	***ARAHAN :*** Bagi soalan-soalan berikut, sila bulatkan nombor yang paling sesuai dengan pandangan anda.
1.	Sejauh manakah diabetes anda menjejaskan kehidupan anda?<br>**0 1 2 3 4 5 6 7 8 9 10**<br>tidak menjejaskan langsung — menjejaskan kehidupan saya dengan teruk
2.	Pada pendapat anda, berapa lamakah diabetes anda akan berterusan?<br>**0 1 2 3 4 5 6 7 8 9 10**<br>tempoh yang amat pendek — selama-lamanya
3.	Pada pendapat anda, sejauh manakah anda dapat mengawal diabetes anda?<br>**0 1 2 3 4 5 6 7 8 9 10**<br>tidak dalam kawalan saya sama sekali — dalam kawalan sepenuh saya
4.	Pada pendapat anda, sejauh manakah rawatan anda membantu mengatasi diabetes anda?<br>**0 1 2 3 4 5 6 7 8 9 10**<br>tidak membantu langsung — amat membantu
5.	Sejauh manakah anda mengalami gejala akibat diabetes anda?<br>**0 1 2 3 4 5 6 7 8 9 10**<br>Tiada gejala langsung — banyak gejala yang teruk
6.	Sejauh manakah anda risau tentang diabetes anda?<br>**0 1 2 3 4 5 6 7 8 9 10**<br>tidak risau langsung — amat risau
7.	Pada pendapat anda, sejauh manakah anda memahami diabetes anda?<br>**0 1 2 3 4 5 6 7 8 9 10**<br>tidak memahami langsung — memahami dengan sangat jelas
8.	Sejauh manakah diabetes anda menjejaskan anda secara emosi? (cth. adakah ia membuatkan anda marah, takut, kecewa atau murung?)<br>**0 1 2 3 4 5 6 7 8 9 10**<br>tidak terjejas langsung secara emosi — amat terjejas secara emosi
9.	Sila senaraikan, tiga faktor paling penting mengikut susunan yang anda percaya telah menyebabkan diabetes anda. Sebab paling penting bagi saya:-<br>**1.** ____________________<br>**2.** ____________________<br>**3.** ____________________

Use of the MBIPQ is protected by Malaysia copyright laws. Permission for use is required.

The Brief Illness Perception Questionnaire (BIPQ)

No.	For the following questions, please circle the number that best corresponds to your views:
1.	How much does your diabetes affect your life?<br>**0 1 2 3 4 5 6 7 8 9 10**<br>no affect at all — severely affects my life
2.	How long do you think your diabetes will continue?<br>**0 1 2 3 4 5 6 7 8 9 10**<br>a very short time — forever
3.	How much control do you feel you have over your diabetes?<br>**0 1 2 3 4 5 6 7 8 9 10**<br>absolutely no control — extreme amount of control
4.	How much do you think your treatment can help your diabetes?<br>**0 1 2 3 4 5 6 7 8 9 10**<br>not at all — extremely helpful
5.	How much do you experience symptoms from your diabetes?<br>**0 1 2 3 4 5 6 7 8 9 10**<br>no symptom at all — many severe symptoms
6.	How concerned are you about your diabetes?<br>**0 1 2 3 4 5 6 7 8 9 10**<br>not at all concerned — extremely concerned
7.	How well do you feel you understand your diabetes?<br>**0 1 2 3 4 5 6 7 8 9 10**<br>don't understand at all — understand very clearly
8.	How much does your diabetes affect you emotionally? (e.g. does it make you angry, scared, upset or depressed?<br>**0 1 2 3 4 5 6 7 8 9 10**<br>no affect at all — severely affects my life
9.	Please list in rank-order the three most important factors that you believe caused <u>your diabetes</u>. The most important causes for me:-<br>**1.** ______________________<br>**2.** ______________________<br>**3.** ______________________
